# A phase II open-label multicenter study of gefitinib in combination with irradiation followed by chemotherapy in patients with inoperable stage III non-small cell lung cancer

**DOI:** 10.18632/oncotarget.12741

**Published:** 2016-10-18

**Authors:** Antonin Levy, Etienne Bardet, Benjamin Lacas, Jean-Pierre Pignon, Julien Adam, Ludovic Lacroix, Xavier Artignan, Pierre Verrelle, Cécile Le Péoux

**Affiliations:** ^1^ Department of Radiation Oncology, Gustave Roussy, Université Paris-Saclay, Institut Thoracique dOncologie (IOT), Villejuif, France; ^2^ INSERM U1030, Molecular Radiotherapy, Gustave Roussy, Université Paris-Saclay, Villejuif, France; ^3^ Univ Paris Sud, Université Paris-Saclay, Le Kremlin-Bicêtre, France; ^4^ Department of Medical Oncology, Institut de Cancérologie de lOuest, Nantes, France; ^5^ Gustave Roussy, Université Paris-Saclay, Department of Biostatistics and Epidemiology, Villejuif, France; ^6^ INSERM U1018, CESP, Université Paris-Sud, Université Paris-Saclay, Villejuif, France; ^7^ Department of Medical Biology and Pathology, Translational Research Laboratory and Biobank (UMS3655 CNRS / US23 INSERM), INSERM Unit U981, Villejuif, France; ^8^ Department of Radiation Oncology, University Hospital Grenoble, Grenoble, France; ^9^ Department of Radiation Oncology, Centre Jean Perrin, Clermont-Ferrand, France; ^10^ Department of Radiation Oncology, St Grégoire Hospital, St Grégoire, France

**Keywords:** iressa, thoracic radiotherapy, lung cancer, phase II trial

## Abstract

**Background:**

Gefitinib is an oral EGFR tyrosine kinase inhibitors which may act as a radiosensitizer.

**Patients and Methods:**

This phase II study evaluated the efficacy of gefitinib 250 mg once daily in combination with thoracic radiotherapy (66 Gy in 6.5 weeks, 2 Gy/day, 5 fractions/week) followed by consolidation chemotherapy (IV cisplatin and vinorelbine) as first line treatment in a population of unselected stage IIIB NSCLC patients according to *EGFR* mutation status.

**Results:**

Due to a low accrual rate in this study, the sample size (*n* = 50) was not reached. Sixteen patients were included in four centers, 50% had adenocarcinoma and 75% were male. Genomic alterations (7 patients studied) retrieved TP53 mutation in 2 patients and no *EGFR* mutation. Four weeks after radiotherapy, 3 patients (19%) had a partial response, 6 (38%) had a stable disease, and 7 had a progression (44%). Median overall survival was 11 months and median progression-free survival was 5 months. At the time of the last contact, 5 patients (31%) were still alive. Main toxicities were gastrointestinal (81%), cutaneous (81%), general (56%), and respiratory (50%). There were 12>G3 adverse events in 7 (47%) patients, and there was one toxic-death during the concomitant period due to an interstitial pneumonitis. There were two possible adverse events-related deaths during the chemotherapy period (pulmonary embolism (*n* = 1) and sudden death after the administration of the 3^rd^ course of chemotherapy (*n* = 1)).

**Conclusion:**

The benefit of Gefitinib-RT could not be confirmed due to premature trial discontinuation. Further evaluation is required, especially in patients with *EGFR* mutated NSCLC.

## INTRODUCTION

The standard treatment for locally advanced unresectable non-small cell lung cancer (NSCLC) is the association of conventional chemotherapy (platinum based doublets) and radiotherapy [1–3]. Outcomes of locally advanced NSCLC however remain poor and new efficient, safe, and more specific treatments are needed. In the last decades, an enhanced understanding of the NSCLC oncogenic molecular pathways has led to the expansion of individualized targeted therapies. Molecular anomalies include KRAS mutation and / or mutation and overexpression of epidermal growth factor receptor (*EGFR*). Mutations in the EGFR tyrosine kinase are observed in approximately in 15% to 62% of patients and are predictors of responsiveness to EGFR tyrosine kinase inhibitors (TKIs) [4–7]. In the metastatic setting, several EGFR TKIs (erlotinib, gefitinib, afatinib) prolong progression-free survival as compared with platinum based chemotherapy doublets in patients with *EGFR* mutated lung cancer [5].

Gefitinib (iressa™, ZD1839) is an oral EGFR TKI indicated for the treatment of adult patients with locally advanced/metastatic NSCLC with activating mutations of *EGFR* [6,7]. Preclinical evidence suggests that gefitinib enhanced the radioresponse of NSCLC cells by suppressing cellular DNA repair [8]. Concomitant use of EGFR inhibitor and radiotherapy has also demonstrated a significantly increased overall survival (OS) as compared with radiotherapy alone in one randomized controlled trial in head and neck cancer [9]. In light of these data, we conducted a phase II trial that aimed to evaluate the efficacy of gefitinib associated with irradiation, followed by chemotherapy in patients with inoperable stage III NSCLC.

## PATIENTS AND METHODS

### Eligibility criteria

Patients (≥18 years) were eligible for inclusion if they had histologically confirmed unresectable non-pretreated stage III NSCLC. Additional inclusion criteria were: at least one measurable lesion according to Response Evaluation Criteria In Solid Tumors (RECIST version 1.0); World Health Organisation (WHO) performance status (PS) of 0 to 2 at inclusion; adequate pulmonary function (forced expiratory volume in 1 second [FEV_1_] is greater than or equal to 1 L, oxygen diffusion capacity of greater than or equal to 40%); pulmonary dose volume histogram V20Gy inferior or equal to 40%; life expectancy of at least 6 months; Female patients could be included if use of secure contraceptive precautions, or post-menopausal. Exclusion criteria were: prior anticancer treatment (including thoracic radiotherapy or anti-EGFR therapy); known hypersensitivity to gefitinib, cisplatin or vinorelbine, or any of the excipients of these product; interstitial lung disease; malignancies diagnosed within the last 5 years; severe coexisting, psychological, or uncontrolled condition; documented or symptomatic metastases, including positive cytology in the case of pleural effusion; pregnancy or breast feeding; concomitant use of inhibitors of CYP3A4; and weight loss of over 15% in the 3 months before the start of the study. ^18^F-fluorodeoxyglucose positron emission tomography (^18^F-FDG PET/CT) and brain magnetic resonance imaging (MRI) were not mandatory for patient inclusion.

### Study design and treatments

This was a phase II, multicenter, open-label, one-arm study in patients with inoperable histologically confirmed stage III NSCLC to explore the efficacy of 250 mg gefitinib administered concurrently with thoracic radiotherapy followed by chemotherapy.

Gefitinib 250 mg was administered orally once daily beginning 7 days before the onset of radiotherapy, until the end of radiotherapy, disease progression, unacceptable toxicity or withdrawal of consent. Conformal thoracic radiotherapy was delivered at the total dose of 66 Gy in 33 daily fractions of 2 Gy, over a 45-day period. There were no time constraints between staging procedures and start of treatment. Four weeks after completion of gefitinib and radiotherapy, patients received 3 cycles of chemotherapy combining intravenous cisplatin (100 mg/m^2^ once every 28 days) and vinorelbine (25 mg/m^2^ once per week for 3 weeks out of 4). We chose cisplatin-vinorelbine doublet for its good efficacy/toxicity profile when delivered sequentially to thoracic radiotherapy [10, 11]. Patients were assessed one month after the completion of the experimental combination and one month after the last chemotherapy cycle with physical examination and imaging studies. The study was approved by the relevant ethics committee/institutional review board and was conducted in compliance with the Declaration of Helsinki as well as good clinical practice guidelines. Written informed consent was obtained from all patients before trial initiation. This study is registered with ClinicalTrials.gov, number *NCT00333294*.

### Molecular analysis

As planned in the study, formalin-fixed, paraffin-embedded material from baseline biopsy was evaluated for Ki67, phospho(p)ERK, pAKT, EGFR, and pEGFR expression using immunohistochemistry (IHC).

Genomic analysis was performed in the accessible samples. The mutational status of specific cancer genes was determined using next generation sequencing based onIon Torrent approach with Ampliseq Cancer Hotspot panelV2 (Lifetechnologie, Darmstadt, Germany) library preparation as previously described [12].

Molecular analysis was an exploratory objective of this study, and it was not mandatory to have enough tissue available for translational research.

### Endpoints and statistical considerations

The primary endpoint was objective response rate (complete response [CR] and partial response [PR]) 4 weeks after completion of gefitinib and irradiation (immediately prior to chemotherapy), based on the RECIST criteria on computed tomography. Secondary endpoints included: objective response rate at study closure, incidence of controlled disease (CR, PR and stable disease [SD]) at study closure, progression-free survival (PFS), duration of response, overall survival (OS), and safety variables (acute and late toxicity graded according to Common Terminology Criteria for Adverse Events v3.0 [CTCAE], adverse events [AEs], drug interruptions, and study drug exposure).

Fleming's method was used to calculate the number of patients required. A sample size of 50 patients was sufficient to have a power of 80% to detect an objective response rate of 60% and 78% as null and alternative hypothesis respectively, with an alpha of 5% (one-sided). If 37 or more responses were observed out of the 50 patients, it would be possible to conclude in favour of the efficacy of the treatment. All patients that were enrolled and received trial drug were considered the intention-to-treat (ITT) population.

OS and PFS were estimated using the Kaplan-Meier method and defined as the time between inclusion and death from any cause for OS, death or tumor progression for PFS, or last follow-up for surviving patients, whichever came first. Median follow-up was calculated according to Schemper's method [13]. Statistical analyses were performed using SAS (version 9.3). All reported p-values are two-sided, and p-values lower than 0.05 were considered significant.

## RESULTS

### Trial conduct

A total of 16 (/22 screened) patients with stage IIIB NSCLC were enrolled in 4 French centers between 09/2004 and 01/2006. Due to a low accrual rate in this study, the sample size was not reached and the study was prematurely closed.

Table 1 lists the main characteristics of the study population. Most patients were males (75%) and the median age was 56 years old (range, 43-70 years). Immunohistochemistry analysis of the tumor was performed for 10 patients (Ki67, pERK, pAKT, EGFR and pEGFR). Two, 2, and 4 patients had positive biopsies for pERK, pAKT, and EGFR, respectively. All patients had low Hirsch scores for pEGFR and median Ki67 proliferation index was 40% (range, 5-90%). Since response to EGFR TKIs relies on mutational status, a genomic analysis was performed on accessible samples. Genomic alterations (*n* = 7 analyzed, others biopsies not available/assessable) retrieved TP53 mutation in 2 patients and no *EGFR* mutation. There were 3 patients without retrieved alteration, and 2 without detectable DNA. Other samples were not accessible or not assessable.

**Table 1:** Patients’ baseline characteristics at inclusion

Characteristics	*N=16 (%)*
Age (years)*	55.5 (43-70)
Gender	
Male	12 (75)
Female	4 (25)
WHO PS score	
0	8 (50)
1	7 (44)
2	1 (6)
Tobacco	
Yes	11 (69)
No	5 (31)
Histology of primary tumor	
Squamous	6 (37)
Adenocarcinoma	8 (50)
Others	2 (13)
TNM classification (Stage IIIB: n=16)	
T2N3M0	3 (19)
T3N2M0	1 (6)
T4N0M0	3 (19)
T4N2M0	5 (31)
T4N3M0	4 (25)
Pulmonary Function Testing*	
FEV1(liter)	2.3 (1.3-3.4)
DLCO (% theo.)	70 (47-103)

* Median (range)

WHO PS: World Health Organisation performance status; N: number of patients; FEV1: Forced Expiratory Volume in one second; DLCO: diffusing capacity for carbon monoxide ; % theo: % of the theoretical value.

### Treatment delivery

Compliance with gefitinib was good: all patients received the entire planned treatment except six interruptions (corresponding to more than 85% of the initial planned dose): four temporary and two patients did not resume the treatment (disease progression: *n* = 1; asthenia: *n* = 1). No dose reduction of gefitinib was allowed. The median duration of gefitinib exposure was 55 days (range, 32-65 days).

All patients but two received the entire planned radiotherapy. The median dose delivered was 60 Gy (range, 40-66 Gy) in 30 fractions (range, 20-33) with a median duration of 48.5 days (range, 30-57 days) with 3D-conformal radiotherapy (*n* = 16; no used intensity modulated radiation therapy [IMRT] or respiratory gating).

Consolidation chemotherapy was delivered 10 patients and 7/10 patients received the three initially planned cycles of cisplatin and vinorelbine. Five/7 patients had a dose reduction (8% of the initially planned dose) from the second cycle.

The median duration of the overall treatment was 132 days (range, 38-165 days). However, even if compliance was good, 10 out of 16 patients (63%) withdrew prematurely from study: one due to an adverse event (G3 hemoglobin decrease during the 2^nd^ cycle of chemotherapy), and others due to cancer related-disease progression or death (two during radiotherapy, four after radiotherapy and before chemotherapy, and three during the chemotherapy period [including one that received the 3 planned cycles]).

### Response and survival

Four weeks after radiotherapy, 3 patients (19%) had a PR, 6 (38%) had a SD and 7 patients (43.8%) had progressive disease (PD). No patients presented with CR. The proportion of patients with objective tumor response was therefore 19%. At study closure, on 14 evaluated patients, there were 4 (29%) PR, one SD (7%; resulting in a disease control rate of 36%), and 9 PD (64%). The median TTP was 148 days (95% confidence interval [CI]: 85-not reached) and recurrence rate was 56% (*n* = 9). No information on patterns of relapse was available.

**Figure 1:**
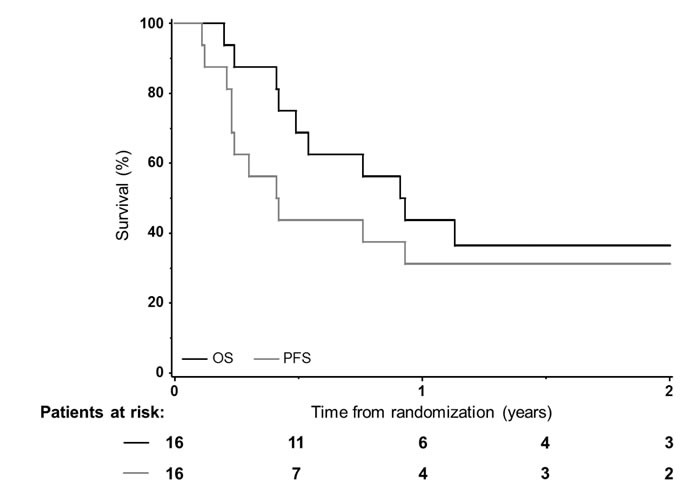
Overall (OS) and progression-free survivals (PFS) among the 16 patients

At the time of the last contact (median follow-up of 2.1 years [range, 1.0-2.4 years]), 5 patients (31%, including three that survived more than two years) were still alive resulting in a median OS of 11 months (95% CI: 5.9-27.7). In this study, 4 patients died during the study and 7 patients died after study withdrawal. Causes of death during the protocol were disease progression (*n* = 1) or adverse events (*n* = 3, cf. below). One-year PFS and OS rates were 31% (95% CI: 14-56%) and 44% (95% CI: 23-67%), respectively (Figure 1). Smoking status did not correlate with survival outcomes.

**Table 2:** Most common AEs (>10 % of patients overall) observed in 16 patients during the combination of gefitinib and radiotherapy (weeks 1-11)

Toxicity	Number of patients N (%)	AEs N
**Gastrointestinal disorders**	**13 (81.3%)**	**34**
Nausea	7 (43.8%)	9
Diarrhea	6 (37.5%)	7
Esophagitis	4 (25.0%)	4
Vomiting	4 (25.0%)	6
Dysphagia	2 (12.5%)	2
Stomatitis	2 (12.5%)	2
**Skin disorders**	**13 (81.3%)**	**16**
Dermatitis	5 (31.3%)	5
Rash	4 (25.0%)	4
Erythema	3 (18.8%)	3
**General disorders**	**9 (56.3%)**	**21**
Chest pain	4 (25.0%)	4
Fatigue	3 (18.8%)	5
Asthenia	2 (12.5%)	3
Mucosal inflammation	2 (12.5%)	2
Pyrexia	2 (12.5%)	4
**Respiratory disorders**	**8 (50.0%)**	**12**
Cough	4 (25.0%)	4
**Investigations**	**7 (43.8%)**	**12**
ALT increased	3 (18.8%)	3
AST increased	3 (18.8%)	3
Weight decreased	2 (12.5%)	2
**Metabolism and nutrition disorders**	**6 (37.5%)**	**8**
Anorexia	4 (25.0%)	4
Dehydration	2 (12.5%)	2
**Nervous system disorders**	**5 (31.3%)**	**7**
Headache	2 (12.5%)	4
**Vascular disorders**	**5 (31.3%)**	**6**
Hypotension	3 (18.8%)	3
**Procedural complications**	**4 (25.0%)**	**4**
Radiodermitis	2 (12.5%)	2
**Cardiac disorders**	**3 (18.8%)**	**5**
**Eye disorders**	**3 (18.8%)**	**3**
**Infections**	**3 (18.8%)**	**3**
**Psychiatric disorders**	**2 (12.5%)**	**2**

AEs: adverse events, N: number of, ALT: Alanine aminotransferase, AST: Aspartate aminotransferase.

### Toxicity

During the experimental combination period, a total of 135 adverse events (AEs) were observed in the 16 patients evaluable, and 12 were > grade (G) 3 (9 G3, two G4, and one G5; cf. Tables 3 & 4). Most frequent AEs (all grades) were: gastrointestinal (81%), cutaneous (81%), general (56%), and respiratory (50%) (Table 2). The most common G3-4 AEs were: gastrointestinal (*n* = 2), general (*n* = 2), hematologic (*n* = 2) and respiratory (*n* = 2) (Table 3). Six patients had at least one serious adverse event (SAE); 2 of these patients had four SAEs. There were three possible adverse events-related deaths: one during the gefitinib-radiotherapy period (interstitial pneumonitis: *n* = 1), and two after (pulmonary embolism at week 18, *n* = 1; sudden death within the hour after the administration of the 3^rd^ course of chemotherapy, *n* = 1).

**Table 3:** Grade 3 or 4 AEs during the combination of gefitinib and radiotherapy (weeks 1 to 11)

Toxicity	Number of patients N (%)	AEs N	Related to gefitinib-RT Number of patients
**Gastrointestinal disorders**	**2 (12.5%)**	**2**	**2**
Esophagitis	2 (12.5%)	2	2
**General disorders**	**2 (12.5%)**	**2**	**2**
Fatigue	2 (12.5%)	2	2
General physical deterioration	1 (6.3%)	1	1
**Investigations**	**2 (12.5%)**	**2**	**1**
ALT increased	1 (6.3%)	1	1
AST increased	1 (6.3%)	1	1
Hemoglobin decreased	1 (6.3%)	1	0
**Respiratory disorders**	**2 (12.5%)**	**2**	**0**
Cough	1 (6.3%)	1	0
Dyspnea exacerbated	1 (6.3%)	1	0
Pneumonia	1 (6.3%)	1	0
**Cardiac disorders**	**1 (6.3%)**	**1**	**1**
Atrioventricular block	1 (6.3%)	1	1
**Procedural complications**	**1 (6.3%)**	**1**	**1**
Interstitial pneumonitis*	1 (6.3%)	1	1
**Metabolism and nutrition disorders**	**1 (6.3%)**	**1**	**0**
Dehydration	1 (6.3%)	1	0
**Vascular disorders**	**1 (6.3%)**	**1**	**0**
Deep vein thrombosis	1 (6.3%)	1	0

AEs: adverse events, N: number of, ALT: Alanine aminotransferase, AST: Aspartate aminotransferase, RT: radiotherapy.

*Grade 5

**Table 4:** Literature overview of concurrent anti-EGFR agents and radiotherapy

Study	Phase	Patients *N*	EGFR+ N (%)*	RT *(Gy)*	Chemotherapy	PFS *(med. m)*	OS *(med. m)*	Toxicity >G4^b^ *(%)*
					*Gefitinib*			
					*Erlotinib*			
					*Cetuximab*			
Ready	II	PR:29 GR:21	13 (26)	66	PR: none GR: Conc Ca Txl	PR: 13.4 GR:9.2	PR: 19 GR: 13	PR:0 GR: G5 pneumonitis (8) G4 neutrop (36)
Niho	II	38	NS	60	Ind CDDP Vin	11.2	28.5	G4 HLE increase (6)
Stinchcombe	II	23	NS	74	Ind Ca Txl Iri Conc Ca Txl	9	16	G4 embolism (4.8) G4 thrombopenia (4.8)
Okamoto	II	9	2 (29)	60	None	NS	NS	None
Center	I	16	NS	70	Conc+Cons Txt	7.1	21	G5 pneumonitis (13)
Rothschild	I	Step 1: 9 Step 2: 5	NS	63	Step 1 : none Step 2 : CDDP	6m: 42.9%	6m: 85.7%	G4 dyspnea (7)
*Current*	*II*	*16*	*0*	*66*	*Cons CDDP Vin*	*5*	*11*	*G5 pneumonitis (6.3)* *G4 pneumonia (6.3)* *G4 dehydration (6.3)*
Lilenbaum	II	75	0	66	Ind Ca nab-Txl	11	17	G4 blood (8) G4 fatigue (1)
Komaki	II	48	4 (8)	63	Conc+Cons Ca Txl	14	36.5	G4 pneumonitis (2)
Socinski	I/II	45	NS	74	Ind+Conc Ca Txl Bev+Cons Bev	10.2	18.4	G4 neutrop (18) G4 esophagitis (2)
Bradley	III	147 110	NS	60 74	Conc+Cons Ca Txl idem	10.8	25	G4 blood (46) G4/5 dyspnea (2) G4 pneumonitis (1) G4 dehydration (2) G4 dysphagia (1)
Blumenschein^a^	II	93	NS	63	Conc+Cons Ca Txl	2y FR : 44.8%	22.7	G5 pneumonitis (2) G5 ARDS (1)
Hallqvist	II	75	NS	68	Ind CDDP Txt	NS	17	G5 pneumonitis (1.4) G4 hypersens (2.8)
Ramalingam	II	40	NS	73.5	Cons Ca Txl	9.3	19.4	G4 infection G4 infusion reaction G4 embolism G4 feb neutrop (9.8)
Govidan	II	53	NS	70	Conc Ca Pem	12.3	25.2	G5 pneumonitis (4) G5 embolism (2)

N: number of; EGFR+ : epidermal growth factor receptor mutation; RT: radiotherapy; Conc: concurrent; Cons: Consolidation (post-RT); med. m: median months; PR: poor risk: GR: good risk; Ca: carboplatin: Txl: paclitaxel; Txt: docetaxel; Vin: vinorelbine; Bev: bevacizumab; Pem: pemetrexed; 6m: 6 month; neutrop: neutropenia; hypersens: hypersensitivity; HLE: hepatic liver enzymes; feb: febrile; ARDS: Acute respiratory distress syndrome; FR: failure rate; y: year; NS: not stated.

*Only genomic analyses are reported

a Only G5 are reported here for this study

b Only the concurrent period is generally reported

## DISCUSSION

This trial was interrupted because of low accrual. The impact of Gefitinib (250 mg daily) in combination with RT on outcomes in patients with locally advanced NSCLC then remains to be determined, especially in *EGFR* mutated patients (no *EGFR* mutated patients in our series). Previous clinical trials assessing gefitinib with irradiation did not include selected patients for *EGFR* mutations (Table 4) [14–19]. In the largest experience reported by Ready et al, stage III NSCLC patients received gefitinib in combination with radiotherapy alone (“poor risk group”: *n* = 21), or with weekly paclitaxel and carboplatin (“good-risk group”: *n* = 39). Poor-risk group outcomes were promising with a median PFS and OS of 13.4 and 19.0 months, respectively (vs. 5 and 11 months in our study). The poorer outcomes reported in our study might be due to the different chemotherapy administration schedule or imaging modality used. Given the time-period of the study, 18FDG-PET and brain MRI were not mandatory for inclusion and may indeed have caused inadequate staging. It may likewise be difficult to differentiate local relapse and computed tomography changes correlating with radiation fibrosis after thoracic irradiation. Modern protocols generally integrate as ^18^F-FDG PET/CT, and a biopsy confirmation if a relapse is suspected. There are also data that EGFR wild-type patients could have a superior local control in comparison with mutated patients, however more distant metastases (no information on pattern of relapse in this study) [20, 21]. In Ready et al's experience, there was no apparent survival difference with *EGFR*-activating mutations (13/45 available tumors, including 2 who had also *T790M* mutations) vs. wild type or *KRAS* mutation [17]. On the other hand, we recently reported in 78 stage IIIA/IIIB NSCLC patients who received irradiation, that selected gene alterations could be associated with a poorer PFS. The *EGFR/ALK* (EML4-anaplastic lymphoma kinase) group and patients with any other mutations (*n* = 28) had a poorer PFS (median 9.6 and 6.0 months; *p* = 0.005) compared to the wild-type group (median = 12.0 months) [22]. Anyway, the question of whether certain gene alterations could be predictive of radio-sensitivity or radio-resistance remains debated.

The combination of gefitinib and radiotherapy was mild. The most common G3-4 AEs were: gastrointestinal (*n* = 2), general (*n* = 2), hematologic (*n* = 2) and respiratory (*n* = 2). In our trial, chemotherapy was delivered sequentially because concomitant administration of chemoradiotherapy and gefitinib could have been much more toxic. The triple combination of bioradiotherapy and chemotherapy may lead to unexpected toxicities [23]. The authors were quite cautious since pulmonary toxicity (interstitial pneumonia or interstitial lung disease) had been reported in stage IV NSCLC, and this may occur more frequently in the Asian population [24, 25]. In other studies, concurrent weekly cisplatin likely enhanced toxicity [12] but weekly paclitaxel-carboplatin did not [17]. Rotschild et al, observed 2 dose-limiting toxicities (DLT) in 9 patients (22.2%) receiving concurrent weekly cisplatin, whereas no DLT occurred in the 5 patients without concomitant chemotherapy. DLT consisted of a G3 pulmonary infection (and thus related to a G4 infection dyspnea), and of a G2 increase in hepatic enzymes.

In our trial, one patient died from an acute interstitial pneumonitis. Others have reported severe interstitial pneumonitis with concurrent irradiation-gefitinib (or other anti-EGFR, Table 4). New available radiotherapy techniques such as IMRT or respiratory gating could help delivering smaller irradiated volumes / sparing organ at risk from higher radiation doses [26, 27]. Though IMRT may be damaging to lung because of low-dose bath. Constraints of normal irradiated lung were, in this study, less tight than recommended nowadays (volume of normal lung minus the planned target volume [PTV] receiving at least 20 Gy (V20) < 40%). This must be emphasized for the conception of new trials, since any loosening in radiotherapy constraints, especially to the lung but also to the heat, may alter the safety of the combination as shown in the recently published RTOG (Radiation Therapy Oncology Group) trial [28].

Of note, concurrent erlotinib and whole brain radiotherapy (WBRT) have also been prospectively studied in brain metastatic NSCLC patients with a good overall response rate (86%) and no increased neurotoxicity [29]. A randomized study still did not show survival advantage for concurrent erlotinib and WBRT followed by maintenance erlotinib in patients with predominantly EGFR wild-type NSCLC as compared to placebo [30].

Additional anti-EGFR treatments [14–19, 28, 31–37] and other newer molecular compounds [38] are tested with concomitant radiation in this setting (Table 4). In the only phase III trial [28], the anti-EGFR cetuximab associated to chemoradiotherapy lead to higher toxic effects but did not increase OS in unselected NSCLC stage III patients. Further experiences evaluating concomitant erlotinib with chemoradiation were also disappointing [31–33]. The RTOG 1306 II trial is currently recruiting patients with mutations in *EGFR* and/or *ALK* fusion arrangement who receive induction erlotinib or crizotinib before thoracic chemoradiotherapy. Finally, as some of the effects of ionizing radiation are now recognized as contributing to antitumor immunity [39, 40], targeting molecules that downregulate the T cell immune response with immunotherapy such as anti-CTLA-4 (cytotoxic T-lymphocyte antigen-4) or anti-PD-1/PD-L1 (programmed death-1 and it ligands) are also currently assessed in patients with locally advanced NSCLC receiving definitive radiotherapy (NCT02125461, NCT02434081, NCT02400814).

## CONCLUSION

In this phase II trial, gefitinib (250mg once daily) in combination with thoracic radiotherapy administered with conventional fractionation at the dose of 66 Gy followed by chemotherapy in previously untreated stage III NSCLC was feasible but lead to substantial toxicities (one toxic death during the concomitant period and two possible adverse events-related deaths during the chemotherapy period). Due to premature trial discontinuation and the absence of patients with *EGFR* mutated NSCLC, our results should be cautiously interpreted. Further investigation is needed to better assess the therapeutic ratio of this combination in trials that take into account modern radiation-delivery techniques and incorporate the biological abnormalities of tumors (*EGFR* mutated NSCLC).

This work was presented at the 16^th^ World Conference on Lung Cancer (WCLC 2015)
